# Identification and profiling of conserved and novel microRNAs from Chinese Qinchuan bovine longissimus thoracis

**DOI:** 10.1186/1471-2164-14-42

**Published:** 2013-01-18

**Authors:** Jiajie Sun, Mijie Li, Zhuanjian Li, Jing Xue, Xianyong Lan, Chunlei Zhang, Chuzhao Lei, Hong Chen

**Affiliations:** 1 College of Animal Science and Technology, Northwest A&F University, Shaanxi Key Laboratory of Molecular Biology for Agriculture, Yangling, Shaanxi, 712100, China; 2 Institute of Cellular and Molecular Biology, Jiangsu Normal University, Xuzhou, Jiangsu, 221116, China

**Keywords:** Bovine, Deep sequencing technology, microRNA, Muscle, Proliferation, Differentiation

## Abstract

**Background:**

MicroRNAs (miRNAs) are a family of ~22 nucleotide small RNA molecules that regulate gene expression by fully or partially binding to their complementary sequences. Recently, a large number of miRNAs and their expression patterns have been identified in various species. However, to date no miRNAs have been reported to modulate muscle development in beef cattle.

**Results:**

Total RNAs from the Chinese Qinchuan bovine longissimus thoracis at fetal and adult stages were used to construct small RNA libraries for Solexa SBS technology sequencing. A total of 15,454,182 clean reads were obtained from the fetal bovine library and 13,558,164 clean reads from the adult bovine library. In total, 521 miRNAs including 104 novel miRNA candidates were identified. Furthermore, the nucleotide bias, base edit and family of the known miRNAs were also analyzed. Based on stem-loop qPCR, 25 high-read miRNAs were detected, and the results showed that bta-miRNA-206, miRNA-1, miRNA-133, miRNAn12, and miRNAn17 were highly expressed in muscle-related tissue or organs, suggesting that these miRNAs may play a role in the development of bovine muscle tissues.

**Conclusions:**

This study confirmed the authenticity of 417 known miRNAs, discovered 104 novel miRNAs in bos taurus, and identified five muscle-specific miRNAs. The identification of novel miRNAs significantly expanded the repertoire of bovine miRNAs and could contribute to further studies on the muscle development of cattle.

## Background

MicroRNAs (miRNAs) are a new class of single-stranded endogenous non-coding small RNA molecules (~22 nucleotides) that bind primarily to the 3′UTR of target mRNAs to repress their translation and accelerate their decay [1], and may regulate up to 30% of genes [2]. The majority of miRNAs are conserved across species and play essential roles in regulating many distinct processes such as brain morphogenesis [3], insulin secretion [4], virus immune defense [5], metabolism, and signal transduction [6]. In addition, several studies have also revealed the significance of miRNAs in myocyte proliferation and differentiation in recent years [7]. For example, miR-1 and miR-133 have distinct roles in modulating skeletal muscle proliferation and differentiation in cultured myoblasts *in vitro* and in *Xenopus laevis* embryos *in vivo*. miR-1 promotes myogenesis by targeting histone deacetylase 4 (HDAC4), a transcriptional repressor of muscle gene expression. In contrast, miR-133 enhances myoblast proliferation by repressing serum response factor (SRF) [8]. MiR-206, miR-1, and miR-133 are muscle specific miRNAs [9]. The transcription of miR-206 is induced by MyoD, which promotes myogenic differentiation [10]. Bone Morphogenetic Protein-2 (BMP-2), which is known to inhibit myogenesis, represses the expression of miR-206 by inhibiting its maturation process [11]. Similarly, overexpression of miR-181 during muscle differentiation is important to promote myogenesis by down-regulating the homeobox protein Hox-A11, an inhibitor of muscle differentiation [12]. MiR-486 has also been shown to induce myoblast differentiation by down-regulating Pax7 [13], while MiR-27b regulates Pax3 protein levels and ensures myogenic differentiation [14]. Recently, the myoblast cell line C2C12 was used for functional analysis of miR-214 *in vitro*[15]. The results showed that miR-214 may target the negative regulators of Myf5, MyoD and myogenin in the corresponding stages of skeletal muscle development *in vivo* to regulate embryonic myogenesis. To date, miRNAs have become one of the most abundant categories of gene regulatory molecules in mammalian species, but the role of individual miRNAs in muscle development is still unknown.

Currently 19,724 mature miRNAs have been discovered from 153 species and deposited in the publicly available miRNA database miRBase (Release 17.0, April 2011) (http://www.mirbase.org). Specifically, the number of miRNAs from bovine species is limited with only 674 reported, compared with 1,921 from human and 1,157 from mouse. Despite the recognized importance of miRNAs in regulating gene expression during development and other biological processes in beef, there has been little information about miRNA expression in cattle. This is surprising given that cattle have tremendous importance not only for food production but as a mammalian model organism for comparative genomics and biological studies [16]. Recent related studies have been conducted to provide insight into the miRNA population present in bovine species by investigating the characteristics, expression pattern and features of their target genes. For instance, 59 distinct miRNAs were identified from bovine adipose tissue and mammary gland in 2006 alone [17]. Bta-mir424 and bta-mir-10b are highly abundant in germinal vesicle oocytes, as well as in early stage embryos (until 16-cell stage) [18]. MiR-196a is a *bona fide* negative regulator of the *newborn ovary homeobox* gene (*NOBOX*) during bovine early embryogenesis [19]. Expression of bovine nucleoplasmin 2 (NPM2) is temporally regulated during early embryogenesis by miR-181a [20]. Approximately 20% of the miRNAs involved in adipogenesis and lipid deposition were identified as being correlated with backfat thickness. The results suggest that miRNAs play a regulatory role in white adipose tissue development in beef [21].

Given the emerging roles of miRNAs in development, identifying the differentially expressed miRNAs is an important first step to investigating the function of miRNAs in the course of bovine growth and development. In this study, we have constructed two small RNA cDNA libraries from Chinese Qinchuan bovine longissimus thoracis at fetal and adult stages. By high throughput sequencing of the small RNA libraries and subsequent bioinformatic analysis, miRNAs in the longissimus thoracis were identified. In addition, the expression patterns of the high-read miRNAs from differential tissues (muscle, heart, liver, lung, kidney, brain, intestine, fat, and spleen) at multiple developmental stages of bovine muscle tissues (fetal, calf, and adult) were evaluated. Elucidation of the expression patterns of different miRNAs among different tissues will contribute to understanding the roles of miRNAs in gene expression regulatory networks for particular biological functions in livestock species.

## Results and discussion

### Tissue collection and high-throughput sequencing of small RNAs

Skeletal muscle is composed of myofibers, intramuscular adipocytes and connective tissue. Myofibers are the structural units of skeletal muscle [22]. In livestock, all muscle fibers are formed during the prenatal stage. Bovine prenatal myogenesis can be briefly divided into three different generations of cells, which appear at around 60, 90, and 110 days of fetal life (post-conception) [23]. In contrast, postnatal skeletal muscle development is mainly due to the increase in muscle fiber size [24], and new muscle fibers are only generated during the adult stage to replace injured muscle fibers [25]. This pattern is significantly different between prenatal and postnatal bovine muscle development. Hence, in this study fetal and adult Chinese Qinchuan bovine longissimus thoracis were collected and two miRNA libraries were constructed for Solexa SBS technology sequencing.

**Figure 1:**
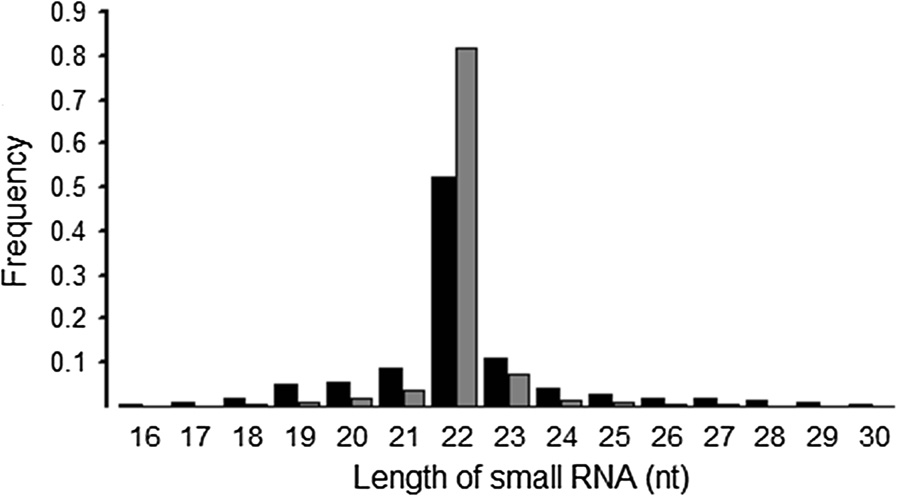
Length distribution of small RNAs in the fetal bovine (black) and adult bovine (gray) libraries.

To identify the small RNAs involved in bovine muscle proliferation and differentiation, total RNAs from bovine longissimus thoracis at fetal and adult stages were used to construct small RNA libraries. We obtained 15,454,182 clean reads from the fetal bovine library and 13,558,164 clean reads from the adult bovine library after deleting some contaminant reads (Table 1). Length distribution analysis showed that most reads ranged from 21 to 23nt. The percentage of the 22nt reads in the total reads was 52.20% for the fetal stage and 81.79% for the adult stage (Figure 1). Next, all Solexa reads were aligned against the latest bovine genome assembly using the program SOAP [26] and 12,298,579 reads of the fetal bovine library were perfectly matched to the bovine genome as well as 12,109,062 reads of the adult bovine library. The most abundant (based on read count) RNA species in both libraries were classified as miRNAs, representing 61.5% of the fetal library and 86.67% of the adult library. The genome-matched small RNA tags were then clustered into several RNA categories (such as known mRNAs, repeats-associated RNA, rRNA, tRNA, snRNA, snoRNA) in the two libraries (Table 2). Additionally, a high percentage of small RNAs were sorted as unknown RNAs (20.59% for fetal bovine and 10.27% for adult bovine).

**Table 1:** Summary of small RNA sequencing date

**Type**	**Fetal bovine muscle tissue**		**Adult bovine muscle tissue**	
	**Count**	**%**	**Count**	**%**
total_read	15941389		13641806	
high_quality	15881408	100%	13606490	100%
adaptor3_null	10834	0.07%	6723	0.05%
insert_null	5218	0.03%	577	0.00%
adaptor5_contaminants	23352	0.15%	5830	0.04%
small_than_18nt	387691	2.44%	35162	0.26%
polyA	131	0.00%	34	0.00%
clean_reads	15454182	97.31%	13558164	99.64%

**Table 2:** Distribution of the genome-mapped sequence reads in small RNA libraries

**Locus class**	**Fetal bovine muscle tissue**		**Adult bovine muscle tissue**	
	**Unique sequences**	**Reads**	**Unique sequences**	**Reads**
Total	688055 (100%)	15454182 (100%)	188906 (100%)	13558164 (0.00%)
miRNA	5073 (0.74%)	9512581 (61.55%)	2492 (1.32%)	11750980 (86.67%)
exon_antisense	734 (0.11%)	893 (0.01%)	105 (0.06%)	186 (0.00%)
exon_sense	72597 (10.55%)	79779 (0.52%)	19608 (10.38%)	21223 (0.16%)
intron_antisense	5624 (0.82%)	8081 (0.05%)	646 (0.34%)	1094 (0.01%)
intron_sense	26620 (3.87%)	34148 (0.22%)	3315 (1.75%)	4799 (0.04%)
rRNA	107912 (15.68%)	2213269 (14.32%)	36130 (19.13%)	324351 (2.39%)
repeat	47495 (6.90%)	95063 (0.62%)	5612 (2.97%)	11922 (0.09%)
scRNA	696 (0.10%)	18870 (0.12%)	235 (0.12%)	3171 (0.02%)
snRNA	5023 (0.73%)	17415 (0.11%)	1062 (0.56%)	2048 (0.02%)
snoRNA	3330 (0.48%)	13414 (0.09%)	667 (0.35%)	2846 (0.02%)
srpRNA	2034 (0.30%)	28132 (0.18%)	611 (0.32%)	2262 (0.02%))
tRNA	24410 (3.55%)	251226 (1.63%)	7375 (3.90%)	41391 (0.31%)
unknown	386507 (56.17%)	3181311 (20.59%)	111048 (58.78%)	1391891 (10.27%)

### Identification of conserved bovine miRNAs

There are currently 662 bta-miRNA precursors and 674 mature bta-miRNAs containing 577 bta-miRNAs, 42 bta-miRNA*, 28 bta-miRNA-5p and 27 bta-miRNA-3p in miRBase. To identify conserved miRNAs in the skeletal muscle tissue of the Qinchuan bovine, the small RNAs with a length of 18–23 nucleotides were Blastn searched against the miRBase 17.0 (miRBase release version V17.0, April 2011). 5,130 unique sequences (9,512,874 reads) were annotated as miRNA candidates in the fetal bovine library as well as 2,508 unique sequences (11,751,013 reads) in the adult bovine library, while the rest were unannotated. The miRNA candidates were then clustered into 407 and 289 categories corresponding to 406 and 299 independent genomic loci in the two libraries according to sequence similarity (Table 3), of which 276 miRNAs overlapped in both libraries (Additional file 1). Each category included multiple homologs, which differed in sequence length by only 1–5 nucleotides. Such homologous sequences with different lengths are thought to be variants produced by various biochemical modifications and by imprecise processing of primary or precursor miRNAs by Drosha and Dicer enzymes. In two cases (bta-mir-126 and bta-mir-424), we discovered that miRNA* were more abundant than corresponding miRNA as evidenced by higher counts of sequence reads originating from the miRNA* arms of the miRNA precursor sequences. Although these cases could be simple annotation artifacts, it is also possible that they reflect the regulated processing of pre-miRNA, which results in preferential use of the different arms of the miRNA precursor [27]. We also identified two miRNAs (bta-mir-151 and bta-mir-455) that demonstrated nearly equal numbers of sequence reads originating from the 5' and 3' arms of the miRNA hairpin precursor. The approximate equivalent expression rates of miRNA and miRNA* (miR-151/151*, 447/680 reads; miR-455/455*, 186/314 reads in the fetal bovine library, (Additional file 2) may be due to the similar 5' end stability, which results in equal incorporation of either strand into the RNA-induced silencing complex (RISC) and protection from degradation [28]. Accordingly, such miRNA genes have also been predicted and validated in chicken [29], drosophila [30] and cow [31]. These miRNA precursors may produce functional molecules on both arms.

**Table 3:** Summary of known miRNA in each sample

	**miR**	miR*	**miR-5p**	**miR-3p**	**pre-miRs**	**Unique matched to pre-miRs**	**Read matched to pre-miRs**
Known miRs	577	42	28	27	662		
Fetal bovine muscle tissue	344	21	21	21	406	5130	9512874
Adult bovine muscle tissue	243	13	16	17	299	2508	11751013

**Figure 2:**
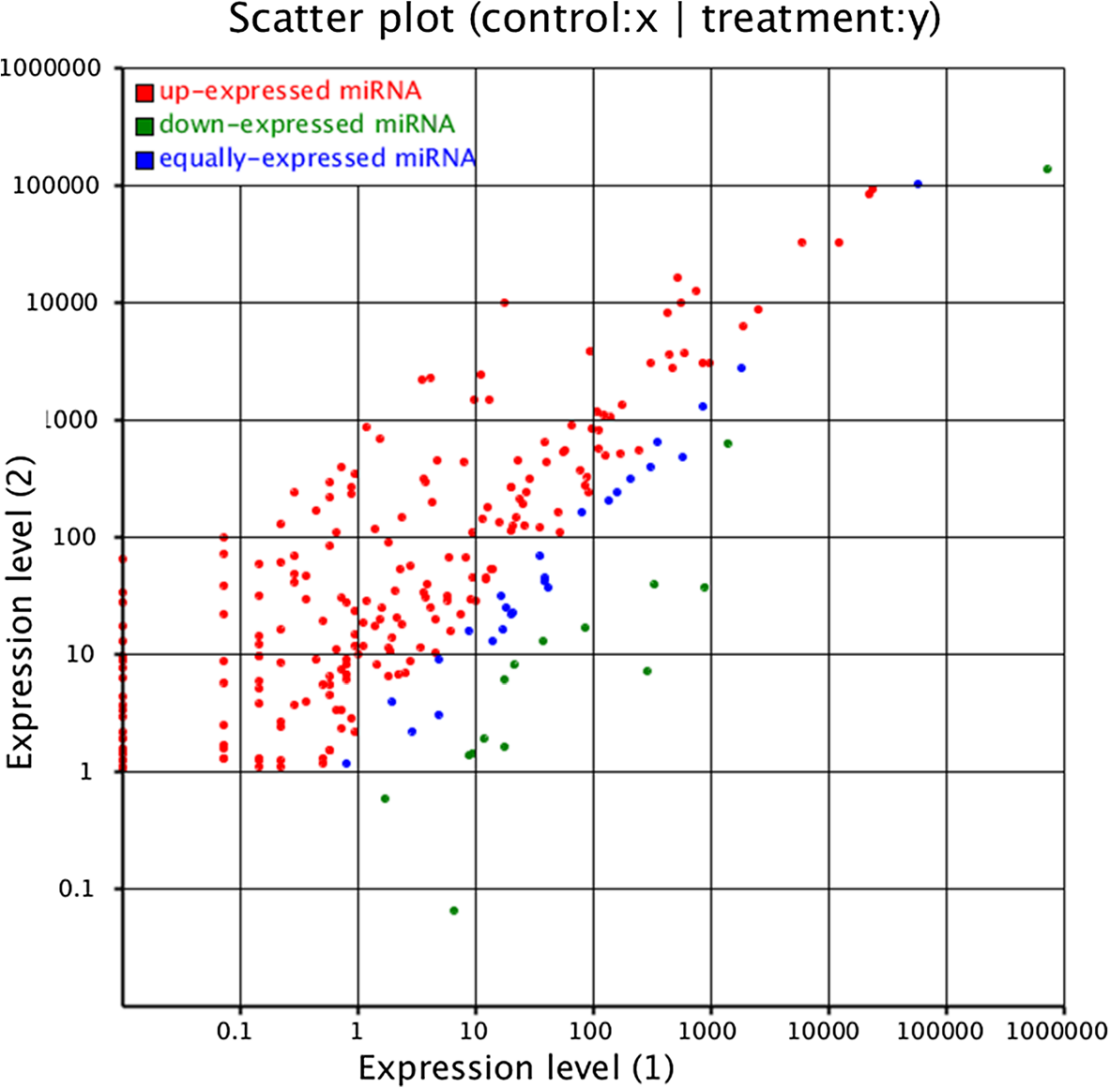
**The differential expression of bovine conserved miRNAs between fetal bovine and adult bovine muscle tissue are shown.** Note: Expression level **(1)**: Expression level of adult bovine muscle tissue; Expression level **(2)**: Expression level of fetal bovine muscle tissue. Each point in the figure represents a miRNA. Red points represent miRNAs with a fold change > 2, blue points represent miRNAs with 1/2 < fold change ≤ 2, green points represent miRNAs with fold change ≤ 1/2.

The expression of known miRNAs in the two samples was demonstrated by plotting Log2-ratio and Scatter Plot (Figure 2). The expression profiles between the two libraries are shown in Additional file 3. The results show that 251 miRNAs consist of 230 up-expressed miRNAs and 21 down-expressed miRNAs. These were significantly different between the two libraries. For example, the expression of miR-103, miR-107, and miR-25 was higher in fetal bovine muscle tissue, in contrast with the patterns shown by miR-1, miR-133a, and miR-29a in adult bovine muscle tissue. This suggests that these miRNAs may affect the development of muscle tissue. Additionally nucleotide bias analysis at each position showed the GC content had a high frequency in the 3rd, 7th, and 19th positions with percentages of 92.64%, 92.80%, and 93.27%, respectively, while it seldom appeared at the 4th, 5th, 8th, 11th, and 20th positions with percentages of 7.49%, 7.30%, 7.26%, 10.25%, and 7.34% in the adult bovine library, respectively. Equally reputable studies have shown the opposite results in the fetal bovine library. In both libraries, nucleotides A + U were distributed mainly in the remaining positions with the exception of the 2nd, 12th, 15th, and 21st positions (Additional file 4). The phenomenon of nucleotide bias might be concerned with the mechanisms of miRNA action, such as binding with the targets for gene regulation. In addition, Zhang et al. reported that the 1st, 9th, and the terminal positions were enriched with U and the 1st and 9th positions were the limits of the “seed region” of a miRNA, which was responsible for targeting mRNAs for gene regulation [32]. We showed a similar result in bovine miRNAs at the first and the end positions, but there was only 7.45% at the ninth position in the adult bovine library. The differences observed between our study and previous studies may have been due to different experimental approaches or differences in the samples (35 species of metazoans compared with Chinese Qinchuan cattle).

Position 2–8 of a mature miRNA is called the seed region, which is highly conserved. The target of a miRNA might be different with the change of nucleotides in this region. In our analysis pipeline, miRNAs that might have base edit can be detected by aligning unannotated sRNA tags with mature miRNAs from miRBase17, allowing one mismatch at a certain position. The results showed that approximately 23.41% in the adult bovine library and 29.06% in the fetal bovine library of the identified miRNA sequences were found to have mismatches that were caused by post-transcriptional modification, and/or RT-PCR, and sequencing errors (Additional files 5 and 6). The prevailing sequence alterations such as 3' terminal A or U additions and A-to-G transitions could result from post-transcriptional modification [33]. In addition, alignment of the identified sequences revealed that end variants were present in most miRNAs, which were apparently generated from the same precursor. Most end variants differed by one or several nucleotides at the 3' end nucleotide and a small percentage of them differed at the 5' end. For example, miR-105b, miR-1185, and miR-122 only had 3' end variants. miR-142a, miR-1271, and miR-138 differed by only one nucleotide in the 5' end, but they had several 3' end variants (Additional file 7). Variation in end (size) in miRNAs may result from the processing of double-stranded RNA (dsRNA), short hairpin RNA (shRNA) and miRNA precursors by Dicer [34,35].

To date, 2,042, 1,281, 791, 289, 360, 103, 247, and 306 conserved miRNAs were identified in human, mouse, chicken, dog, horse, sheep, zebrafish and wild boar, respectively http://www.mirbase.org/. In our study, further analysis identified a total of 289 and 407 conserved miRNAs that belonged to 200 miRNA families in adult and fetal bovine libraries. The identified miRNA families have been shown to be conserved in a variety of species. For example, let-7, miR-1, miR-34, miR-9, and miR-25 families have been found in 64, 61, 56, 65 and 62 species, respectively, while miR-2363, miR-2384, miR-2404, miR-2424, and miR-409 families have only been detected in *bos taurus* (Additional file 8). This may suggest a species-specific expression profile for miRNAs. The largest miRNA family size identified was miR-2284, which consisted of 12 members, and let-7, miR-30, and miR-181/376 possessed 9, 7, and 4 members, respectively; whereas other miRNA families such as miR-1, miR-31, miR-93, and miR-206 had only one member (Additional file 1). The finding that most members of conserved miRNA families were expressed in the bovine longissimus thoracis supports the idea that regulatory or functional diversification has occurred [36,37]. Different family members also displayed drastically different expression levels. For example, the abundance of the miR-2284 family varied from 1 read (bta-miR-2284a) to 46,548 reads (bta-miR-2284x) with deep sequencing. This was also the case for some other miRNA families, such as bta-let-7 (from 6 to 1,434,682 reads), bta-miR-30 (from 34 to 12,681 reads) and bta-miR-181 (from 376 to 20,258 reads). However, the expression levels of some miRNA families were similar, such as miR-15 in which 246 and 276 reads were detected, respectively. The existence of a dominant member in a miRNA family may suggest that the regulatory role of this family was performed by the dominant member at the developmental time when the samples were collected for RNA extraction. Abundance comparisons of different members in a miRNA family may provide valuable information on the role that miRNAs play in that specific stage of bovine development.

### Identification of novel bovine miRNAs

The characteristic hairpin structure of a miRNA precursor can be used to predict novel miRNA. Prediction software Mireap http://sourceforge.net/projects/mireap/ was developed to predict novel miRNA by exploring the secondary structure, the Dicer cleavage site and the minimum free energy of the unannotated small RNA tags that could be mapped to a genome. The following criteria [38] were used for screening the candidates for potential miRNAs or pre-miRNAs: (1) Pre-miRNA sequences can fold into an appropriate hairpin secondary structure that contains the ~22nt mature miRNA sequence within one arm of the hairpin. (2) miRNA precursors with secondary structures had higher negative minimal free energies (MFEs) and minimal free energy indexes (MFEIs) than other different types of RNAs. (3) miRNA had an AU content of 30–70%. (4) miRNA had less than six mismatches with the opposite miRNA* sequence in the other arm. (5) No loop or break in miRNA sequences was allowed. Based on Solexa sequencing, we identified 104 novel bovine miRNAs, which corresponded to 145 genomic loci. Thirty-six novel miRNAs were in the adult bovine library and 92 were in the fetal bovine library, of which 24 overlapped in both libraries (Additional file 9). In addition, an examination of pre-miRNAs and other RNAs (tRNA, rRNA, and mRNA) revealed that miRNAs were significantly different from other RNAs [39]. Specifically, more than 90% of miRNA precursors have an MFEI greater than 0.85, significantly higher than tRNAs (0.64), rRNAs (0.59), or mRNAs (0.65). The results suggested that the MFEI can easily be used to distinguish miRNA from other non-coding and coding RNAs. This provides a more precise criterion to predict miRNAs using computational approaches, and in our database 122 had a MFEI greater than 0.85. Remarkably, the read number for each novel miRNA was much lower than that for the majority of conserved miRNAs. For instance, bta-miRn70 with the highest read number and bta-miRn96 with the lowest were both novel miRNAs, and the total reads were only 541,928 and 5, respectively. Recently, similar results in pigs [40] and even in maize [41] have also been reported.

### Validation of related bovine miRNAs

**Figure 3:**
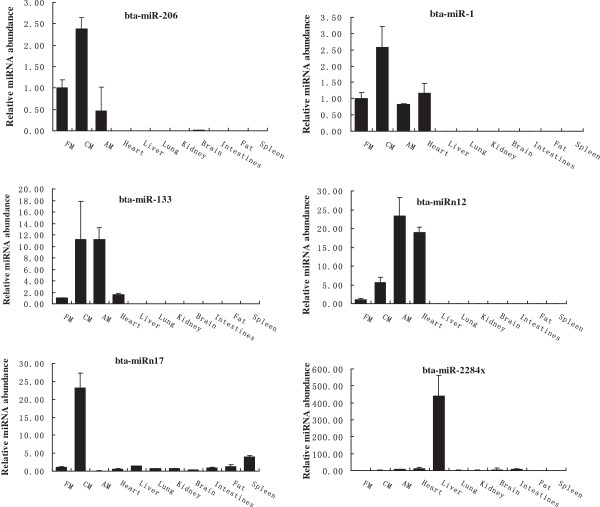
**The expression of miRNAs in bovine muscle-related tissue or organs were detected by RT-qPCR.** Note: FM: fetal bovine muscle tissue; CM: calf bovine muscle tissue; AM: adult bovine muscle tissue.

The miRNA expression patterns in different tissues have been investigated in cattle [18,42]. In beef cattle, miR-9 and miR-124 in the brain, miR-122 in the liver, and miR-1, miR-133a, and miR-206 in muscle are all tissue-specific [42]. For validation and identification of muscle-related miRNAs in Qinchuan cattle, stem-loop qPCR [43] analysis of miRNA expression was performed in fetal, calf and adult skeletal muscle, heart, liver, lung, kidney, brain, intestines, fat and spleen. In the present study, we randomly picked out several high-read miRNAs, including a total of 12 conserved and 13 novel miRNAs. Comparison of miRNA expression profiles among tissues revealed that miR-2284x in liver, and miRNA-206, miRNA-1, miRNA-133, miRn12, and miRn17 in muscle-related tissue or organs (skeletal muscle, heart, intestines) were highly expressed (Figure 3). In contrast, five conserved miRNAs (bta-miRNA-543, miRNA-432, let-7i, miRNA-320, and miRNA-152) and seven novel miRNAs (bta-miRn16, miRn18, miRn33, miRn65, miRn70, miRn73, and miRn73) could be quantified from all tissues and several of them (*e.g.*, bta-miRNA-320, miRn33 and miRn65) were relatively consistent across all nine tissue types. In addition, only bta-miRNA-29a, miRNA-495, and miRn3 were not expressed in fat, while miRn3 and miRn35 were detected in all tissues except spleen. Another two miRNAs (bta-miRNA-487b and miRn2) were not found in fat and spleen tissues (Additional file 10). In the present study, the bta-miR-487b, miR-495, miRn2, and miRn3 were not identified in the bovine calf and/or adult muscles, but were highly expressed in the fetal stage and in other differentiated tissues. It could be that some protein expression of muscle-specific genes from the tested individuals at postnatal days represses the expression of these miRNAs by inhibiting their maturation process. This is similar to the results between bone morphogenetic proteins-2 (BMP-2) and miR-206 [11]. The expression patterns and level of 25 miRNAs in all tested bovine tissues suggests that these miRNA may be more relevant to the highly conserved biological process in mammalians. Further study to discover their regulatory functions is needed.

To further explore the muscle-specific miRNAs involved in the muscle development of the cattle, we performed quantitative analysis of the miRNAs in fetal (day 90 bovine embryos), calf (3-day-old) and adult (2-year-old) bovine longissimus thoracis. The results showed that expression of bta-miRNA-133 and miRn12 was increased in the muscle tissues from day 90 bovine embryos to 2-year-olds, respectively. However, the expression levels of bta-miRNA-206, miRNA-1, and miRn17 did not change between day 90 bovine embryo and 2-year muscle tissues, but significantly increased in the calf muscle tissue (Figure 3). Previous studies *in vitro* have shown that miR-1, miR-133, and miR-206 can target multiple muscle-development-related genes. Specifically, muscle-specific miR-206, which is directly activated by MyoD, can target sequences in the *Fstl1* and *Utrn* gene and these sequences are sufficient to suppress gene expression in the presence of miR-206 [8]. miR-1 promotes myogenesis by targeting HDAC4, a transcriptional repressor of muscle gene expression. In contrast, miR-133 enhances myoblast proliferation by repressing SRF [44]. Also, miR-1 and miR-206 regulate Pax7 directly. Inhibition of these two substantially enhances satellite cell proliferation and increases Pax7 protein levels *in vivo*[45]. Although the bovine-specific target genes of miRNA-206, miRNA-1, miRNA-133, miRn12, and miRn17 are not known, their consistent expression pattern and high conservation indicate that they are also likely to play roles in the development of bovine muscle tissues.

## Conclusions

We have identified 417 known miRNAs and 104 novel miRNAs in longissimus thoracis from fetal and adult Qinchuan bovine using deep sequencing technologies. This study expands the repertoire of bovine miRNAs and could initiate further study in the muscle development of cattle. In addition, the miRNA expression patterns among nine tissues in beef cattle showed that most miRNAs are ubiquitously expressed, suggesting that these miRNAs may play a role in a broad range of biological processes in various tissues. However, we have also identified some muscle-specific miRNAs, suggesting that these miRNAs are likely to play a role in the development of bovine muscle tissues and could be potential molecular markers for genetics and breeding.

## Methods

### Ethics statement

All animals in this study were maintained according to the No. 5 proclamation of the Ministry of Agriculture, P. R. China. Sample collection was approved by the Animal Care Commission of the College of Animal Science and Technology, Northwest A&F University. Bovine embryos of slaughtered cows were collected from Tumen abattoir, a local slaughterhouse of Xi'An, P.R. China. A newborn Qinchuan calf and adult Qinchuan cattle were obtained from Meixian Qinbao Co., Ltd.

### Tissue collection and high-throughput sequencing

Day 90 (d90) bovine embryos (gestation period 280 days) were collected into sterile physiological saline immediately after removal from the reproductive tract of slaughtered cows at a local abattoir. Fetal age was estimated based on crown-rump length [46]. Bovine tissue samples including the longissimus thoracis, heart, liver, lung, kidney, cortex (brain), small intestine, fat and spleen were collected from fetal, calf and adult Chinese Qinchuan bovine. These tissues were snap-frozen in liquid nitrogen and stored at −80°C until use. In this study, two miRNA libraries were constructed. Total RNAs were extracted from three fetal and three adult Chinese Qinchuan bovine longissimus thoracis were pooled, respectively. Subsequently, low molecular weight RNAs were separated by 15% polyacrylamide gel electrophoresis (PAGE), and RNA molecules in the range of 18–30nt were enriched and ligated with proprietary adapters to the 5^′^ and 3^′^ termini. A reverse transcription reaction followed by low cycle PCR was performed to obtain sufficient product for Solexa technology (Beijing Genomics Institute, China).

### Small RNA sequence analysis

After clearing away the 3′ adaptor sequence, removal of redundancy and reads smaller than 18nt, the clean reads were screened against and mapped to the latest bovine genome assembly http://hgdownload.cse.ucsc.edu/goldenPath/bosTau4/bigZips/bosTau4.fa.gz using the program SOAP [26]. To identify sequences originating from protein-coding genes, repeats, rRNA, tRNA, snRNA, and snoRNA, we used bovine mRNA http://hgdownload.cse.ucsc.edu/goldenPath/bosTau4/database/refGene.txt.gz and CDS http://hgdownload.cse.ucsc.edu/goldenPath/bosTau4/bigZips/refMrna.fa.gz, RepeatMasker http://www.repeatmasker.org and Sanger Rfam data (version 10.1). Subsequently, the remaining reads were searched against the Sanger miRBase (version 18.0) to identify the conserved miRNAs. Only those small RNAs whose mature and precursor sequences perfectly matched known bovine miRNAs in miRBase were considered to be conserved miRNAs. To discover potential novel miRNA precursor sequences, unique sequences that have more than 10 hits to the genome or match to known non-coding RNAs were removed. Then the flanking sequences (150 nt upstream and downstream) of each unique sequence were extracted for secondary structure analysis with Mfold http://www.bioinfo.rpi.edu/applications/mfold and then evaluated by Mireap http://sourceforge.net/projects/mireap/. Specifically, the miRNA candidates that passed Mireap were deemed as highly probable if their corresponding miRNA*s were also found in the small RNA libraries. After prediction, the resulting potential miRNA loci were examined carefully based on the distribution and numbers of small RNAs on the entire precursor regions. Those sequences residing in the stem region of the stem-loop structure and ranging between 20–22nt with free energy hybridization lower than −20 kcal/mol were considered [47].

### MicroRNA expression analysis

Comparison of the known miRNA expression between two samples was conducted to find out the differentially expressed miRNAs. The expression of miRNA was shown in two samples by plotting a Log2-ratio figure and a Scatter Plot. The procedures were shown as below: (1) Normalize the expression of miRNA in two samples (fetal and adult longissimus thoracis) to get the expression of transcript per million. Normalized expression (NE) = Actual miRNA count/Total count of clean reads. (2) Calculate fold-change and P-value from the normalized expression. Then generate the log2-ratio plot and scatter plot. Fold-change formula: Fold_change = log2 (fetal NE/adult NE). P-value formula:

$$P\left(x|y\right)={\left(\frac{N^{2}}{N_{1}}\right)}^{y}\frac{\left(x+y\right)!}{x!y!{\left(1+\frac{N_{2}}{N_{1}}\right)}^{\left(x+y+1\right)}}\begin{array}{c} C\left(y\leq y_{\min}|x\right)=\sum_{y=0}^{y\leq y_{\min}}p\left(y|x\right)\\ D\left(y\geq y_{\text{man}}|x\right)=\sum_{y\geq y_{\max}}^{\infty }p\left(y|x\right) \end{array}$$

The x and y represented normalized expression levels, and the N1 and N2 represented total count of clean reads of a given miRNA in small RNA libraries of the fetal and adult stage, respectively. Stem-loop real-time reverse transcription polymerase chain reaction (RT-PCR) with SYBR Green was used for the analysis of miRNA expression [48]. Total RNA (1 μg from tested tissues) was converted to cDNA with a RT primer mixture (250 nM) using PrimeScript® RT reagent Kit with gDNA Eraser (TaKaRa, Dalian, China). The cDNA was then used for real-time PCR quantification of miRNA using the miRNA specific primer and the universal primer. The bovine ribosomal protein S18 (RPS18) (GenBank NO. NM_001033614.1) gene was used as an endogenous control. The primers for miRNAs and the control gene are listed in Additional file 11. Real-time quantitative PCR was performed using a Bio-Rad CFX 96™ Real Time Detection System and SYBR Green PCR Master Mix (TaKaRa, Dalian, China) in a 20 μl reaction. All reactions were carried out in triplicate. The PCR mix included 100 ng cDNA for each miRNA, 0.4 μM forward and reverse primers, and 10 μl 2 × SYBR Green PCR Master Mix. The cycle conditions were as follows: 95°C for 30 s, followed by 40 cycles of 95°C for 10 s, 60°C for 10 s, and 68°C for 20 s. The threshold cycle (Ct) was defined as the cycle number at which the fluorescence intensity passed a predetermined threshold. The quantification of each miRNA relative to RPS18 gene was calculated using the equation: N = 2^-ΔΔCt^.

## Competing interests

The authors declare that they have no competing interests.

## Authors’ contributions

JJS gathered samples, conceived the report, participated in its design, performed data analysis, interpreted results and drafted the manuscript. MJL and ZJL participated in intellectual discussion. JX contributed to gathering samples. XYL, CLZ, CZL and HC provided guidance on experimental design and funding. All authors read and approved the final manuscript.

## Supplementary Material

Additional file 1 Bovine conserved miRNAs. Click here for file

Additional file 2 The annotated miRNA and its miRNA* in the fetal bovine library. Click here for file

Additional file 3 The known miRNAs expression profiles between two libraries. Click here for file

Additional file 4 **Nucleotide bias at each position of sRNA tags.** Note: miRNA nucleotide bias at each position of adult bovine muscle tissue (A) and fetal bovine muscle tissue (B). Click here for file

Additional file 5 Summary of base edit in adult bovine library. Click here for file

Additional file 6 Summary of base edit in fetal bovine library. Click here for file

Additional file 7 End variants of the fetal bovine library identified in this study. Click here for file

Additional file 8 Family analyses of known miRNAs. Click here for file

Additional file 9 Novel miRNAs identified in this study. Click here for file

Additional file 10 **The expression of miRNAs in bovine tissues and organs were detected by RT-qPCR.** Note: FM: fetal bovine muscle tissue; CM: calf bovine muscle tissue; AM: adult bovine muscle tissue. Click here for file

Additional file 11 Stem-loop RT-PCR Primer. Click here for file
