# Supplementary material for: Identification and profiling of conserved and novel microRNAs from Chinese Qinchuan bovine longissimus thoracis
Source: BMC Genomics. 2013 Jan 18;14:42. doi: 10.1186/1471-2164-14-42 (PMC3563516; doi:10.1186/1471-2164-14-42)
Supplement: Additional file 4 — Nucleotide bias at each position of sRNA tags. Note: miRNA nucleotide bias at each position of adult bovine muscle tissue (A) and fetal bovine muscle tissue (B). [file 1471-2164-14-42-S4.doc]

(A)

[
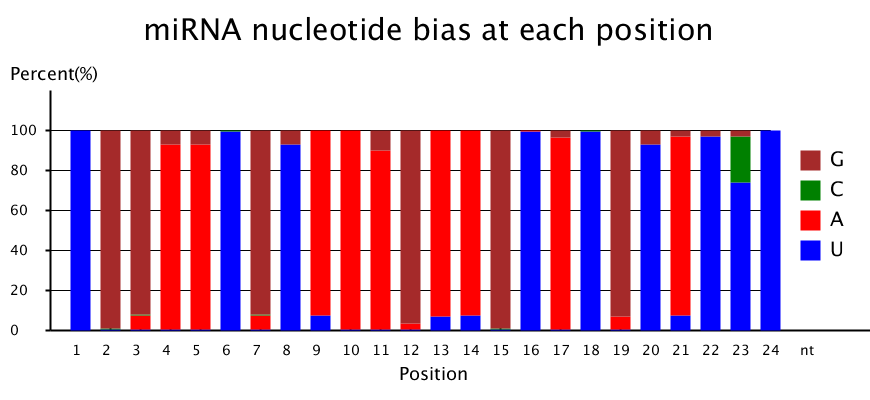
](file:///D:%5C博士实验%5C博士%5CmicoRNA%5C测序结果%5CBGI_SmallRNA_report%5CBGI_SmallRNA_report%5CFiles%5CBGI_graph%5Cmatch_hairpin%5C1%5Chairpin_miRNA_base_bias.png) (B)

[
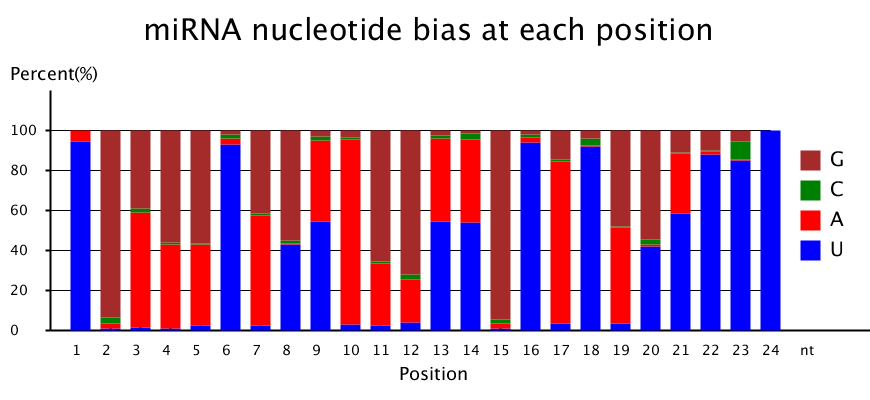
](file:///D:%5C博士实验%5C博士%5CmicoRNA%5C测序结果%5CBGI_SmallRNA_report%5CBGI_SmallRNA_report%5CFiles%5CBGI_graph%5Cmatch_hairpin%5C2%5Chairpin_miRNA_base_bias.png)**Additional file 4** Nucleotide bias at each position of sRNA tags. *Note:* miRNA nucleotide bias at each position of adult bovine muscle tissue (A) and fetal bovine muscle tissue (B), respectively.
