# Supplementary material for: Identification and profiling of conserved and novel microRNAs from Chinese Qinchuan bovine longissimus thoracis
Source: BMC Genomics. 2013 Jan 18;14:42. doi: 10.1186/1471-2164-14-42 (PMC3563516; doi:10.1186/1471-2164-14-42)
Supplement: Additional file 10 — The expression of miRNAs in bovine tissues and organs were detected by RT-qPCR. Note: FM: fetal bovine muscle tissue; CM: calf bovine muscle tissue; AM: adult bovine muscle tissue. [file 1471-2164-14-42-S10.doc]

**Additional file 10** The expression of miRNAs in tissues and organs of the bovine were detected by RT-qPCR. *Note:* FM: fetal bovine muscle tissue; CM: calf bovine muscle tissue; AM: adult bovine muscle tissue.
